# Exploring HIV self-testing as an early detection strategy among female sex workers in Waliso town, Ethiopia: a community based cross sectional study

**DOI:** 10.1186/s12981-025-00729-5

**Published:** 2025-03-18

**Authors:** Kotu Merga, Tomas Benti, Gada Edea, Firaol Regea, Tesfu Zewdu, Hailu Merga

**Affiliations:** 1 Waliso town Health office, Waliso, Oromia region Ethiopia; 2 Department of Public Health, Madda Walabu University, Bale Goba, Ethiopia; 3 Department of Nursing, School of Health sciences, Ambo University Waliso campus, Waliso, Ethiopia; 4 Department of Epidemiology, Institute of Health, Jimma University, P.O. Box 378, Jimma, Ethiopia

**Keywords:** HIV self-testing, Associated factor, Female sex worker, Waliso, Ethiopia

## Abstract

**Background:**

The burden of HIV infection among key population like female sex workers (FSW) is higher and challenges the prevention and control of the virus compared to other population groups. HIV self-testing allows people to test themselves discreetly and conveniently and may provide opportunities to people not currently reached by existing HIV testing and counseling services. Hence, this study aimed to assess the magnitude of HIV Self-Testing (HIVST) and associated factors among Female sex workers in Waliso Town in Central Ethiopia.

**Method:**

A community-based Cross-sectional study was conducted from 1 October–November 30, 2023, among female sex workers using snowball sampling. A total of 400 participants were included in the study. Data was collected using pre-tested, structured self-administered, and interviewer-administered questionnaires using face-to-face interviews. A binary logistic regression model was fitted using SPSS version 26 to identify factors associated with HIV self-testing. Adjusted Odds Ratio (AOR), 95% confidence interval, and a *p*-value < 0.05 was used to judge the statistically significant variables.

**Results:**

The prevalence of HIV self-testing among female sex workers in Waliso town was found to be 37% (95% CI: 32, 42). Education status (attended high school and above) (AOR = 7.62[95% CI 2.55,24.67], marital status (divorced) (AOR = 2.1[95% CI 1.23,3.6], those whose both parents dead (AOR = 2.72[95% CI 1.4,5.28] and before sex whether they asked their partner test status (AOR = 0.17[95% CI 0.07,0.37] were statistically significant.

**Conclusion:**

This study revealed that HIV self-tests among female sex workers were lower than the 95% national target. Education status, marital status, parent`s living status and knowing the partner HIV status before sex were found to be predictors of HIV self-test. Our findings underscore the need to develop evidence-based strategies to improve HIV testing uptake by FSWs and improve community-based services.

## Background

The Human Immunodeficiency Virus (HIV) is a significant global public health issue, affecting mostly Key population like female sex workers due to their specific higher-risk behaviours. Because of marginalization, discrimination, and criminalization, the prevalence of HIV among these population were 3% [1]. HIV prevalence in sub-Saharan Africa is at least three times higher than the general population, posing a high burden. Africa is far from the UNAIDS 95-95-95 target by 2030 [2, 3].

To increase engagement in HIV prevention services and lower the risk of transmission, the World Health Organization (WHO) recommends female sex workers (FSWs) to get tested for HIV frequently. Because, it serves as both the gateway to treatment and the provision of support services [4]. HIV Self testing is a procedure that enables individuals to perform an HIV test on themselves in a private setting by collecting specimens, testing them for HIV antibodies, interpreting the results, and seeking confirmation by visiting a medical center. It is a safe, accurate, and confidential option to reach high-risk populations including commercial sex-workers [5, 6].

Female sex workers are particularly vulnerable to HIV infection and transmission because of their involvement in risky sexual behaviors and concurrent sexual engagements. They face several obstacles to accessing health services, including substance abuse, homelessness, a lack of financial means, social isolation, victimization, and mental health issues [7]. On the other hand, the difficulty in getting diagnostic tests is also one of the challenges [8].

There is variability in accepting of HIVST in sub-Saharan African Countries among female sex workers [9]. A finding from Morocco showed a very high rate of acceptability of the HIVST (90.6%) [10] while the finding from Ethiopia (59.3%) [11] and South Africa (32.5%) [12] reported low prevalence. The reason for this disparity needs further investigation. In addition, although HIVST is recommended as it decreases discrimination and stigma, its utilization is less known in Ethiopia in general and in the specific study area in particular. Hence, this study aimed to assess the magnitude of HIV Self-Testing and associated factors among female sex workers Waliso Town, central Ethiopia.

## Methods and materials

### Study design and setting

A community-based Cross-sectional study design was conducted in Waliso town, southwest Shewa zone, Oromia regional state, central Ethiopia, from October 1, 2023, to November 30, 2023. Waliso town is located approximately 114 km away from Addis Ababa, the Ethiopian capital. It has a total population of 65,322, of whom 33,014 are male and 32,308 are women [13]. There were two hospitals, one public, one Non-Governmental Organization, two health centers, eighteen medium clinics, two specialty clinics, two small clinics, one Family Guidance Association clinic, two dental clinics, seventeen drug stores, and pharmacies that provide basic health services for urban populations. Waliso Town was one of the areas in the Oromia region where a high caseload of HIV/AIDS patients is receiving treatment and care services. The data from the town health office showed that there were about 840 female sex workers in the town (Waliso town report 2023).

### Sampling and sample size

All Female sex workers living in Waliso town were the source population and selected female sex workers who were volunteers during the study period were the study population. Respondents who were unable to provide consent on their own either due to ill health, altered mental state, or any other reason were excluded from the study.

The sample size was determined using EpiInfo (CDC, version 7.2, Atlanta, GA, USA) software with the following assumptions: 80% power, 95% confidence interval, 21% proportion of individuals having Knowledge of HIV self-test and 1.998 Adjusted Odds Ratio from the previous study [11]. Then, by adding 10% non-response rates, the final sample size became 400 study participants.

**Fig. 1:**
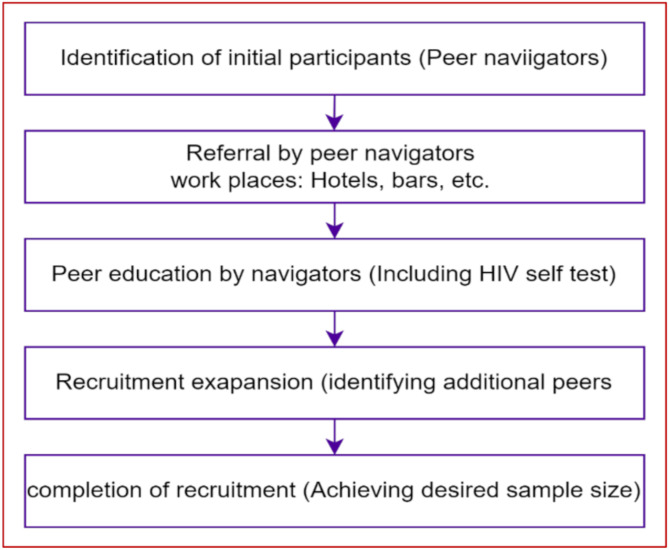
Sample procedure flow chart

To recruit study participants, the snowball sampling technique was used. The initial information was taken from the self-identified female sex worker’s working as a peer navigator recruited by Waliso town health office. Peer navigators at their workplace of female sex workers referred them to the key population service package for peer education on HIV testing, family planning, Sexually Transmitted Infection screening, condom service, gender-based violence service, and HIV Pre-Exposure Prophylaxis service from hotels, bars, local drinking houses, shisha houses, and cafeterias found in the town and surroundings. Discussions were made with them to indicate other female sex workers peers who can participate in the study. A similar procedure was followed until the desired sample size was fulfilled (Fig. 1).

### Measurements

#### HIV self-testing

is a process of HIV testing in which an individual collects her specimen (oral fluid) and then performs an HIV test and interprets the result either by herself or by helping a peer navigator. With the recent release of the OraQuick oral test, self-testing for HIV has become a more practical and accessible option. The HIV-1/2 kit is also one of the state-of-the-art self-test kits recommended by the WHO, as it has been proven highly accurate when used by laypeople.

#### Female sex workers (FSWs)

were defined in this study as females who regularly sex for money in hotels, drinking establishments, nightclubs, local drinking houses, and shisha houses. Knowledge about HIV self-testing was assessed by asking them questions specifically developed to check how to self-test.

#### Knowledge

Knowledge of participants’ HIV knowledge was assessed with twenty-three knowledge questions with yes and no response options. Participants who provided at least more than twelve correct answers to these questions were defined as having a higher level of HIV knowledge, and participants who provided less than twelve correct answers to these questions were defined as having a lower level of HIV knowledge.

### Data collection and quality control

The data collection tool was developed after reviewing different relevant literature [10–12]. It was initially developed in English and then translated Afaan Oromo and Amharic languages and then back translated to English by experts to check its consistency. Then, the structured interviewer-administered questionnaire was used for data collection. Two bachelor diploma holder nurses who had experience in data collection and spoke Afaan Oromo and Amharic were recruited for data collection. One bachelor degree-holder nurse was supervising the data collection. To ensure the quality of the data, the tool was pretested at nearby Tulu Bolo town. The data collectors and supervisor were trained for one day on the objective of the study, procedures, techniques, and approaches of data collection. Daily, the collected data were reviewed and checked for completeness and consistency.

### Data processing and analysis

The strengthening the reporting of observational studies in epidemiology (STROBE) checklist was used to analyze and report data [14]. Before data analysis, the data was cleaned, coded, and entered into the EpiInfo version 7.2 (CDC, version 7.2, Atlanta, GA, USA) statistical software application. Then cleaned data was exported to SPSS (IBM SPSS Statistics, Version 26, Armonk, NY, USA) for further data management and analysis. Descriptive analysis using frequency, mean, standard deviation, and percentages were done. Bivariable logistic regression analysis was done to identify candidates for multivariable logistic regression analysis. Variables with a *p*-value less than 0.25 on the bivariable logistic regression model were entered into the multivariable logistic regression model to identify associated factors of HIV-Self testing among female sex workers. The goodness of the model was assessed by using the Hosmer and Lemeshow test. Multicollinearity was checked by using the variance inflation factor (VIF) and tolerance test. The results of multivariable logistic regression analysis were presented using an Adjusted Odds Ratio (AOR) with a corresponding 95% CI and statistical significance was declared at *p*-value < 0.05.

## Results

### Socio-demographic characteristics of respondents

Of the total 400 respondents, about half 189 (47.3%) were aged 18–24 years and their mean (SD) was 25.02 (SD ± 4.24). More than three fourth 75.5% of them were Orthodox in their religion and more than half 229 (57.3%) attended primary education. With regards to their marital status, nearly half 191 (47.8%) of them were single. The majorities 246 (61.5%) place of birth were urban (Table 1).

**Table 1:** Socio-Demographic characteristics of female sex workers at Waliso town, Ethiopia,2024

Variables	Category	Frequency	Percent (%)
Age	< 18 years	5	1.3
	18–24 years	189	47.3
	25–30 years	171	42.8
	31 + years	35	8.8
Religion	Orthodox	302	75.5
	Muslim	59	14.8
	Protestant	32	8
	Others**	7	1.8
Educational status	Unable to read and write	23	5.8
	Able to read and write	73	18.3
	Primary education	229	57.3
	Secondary school and above	75	18.8
Marital status	Single	191	47.8
	Divorced	155	38.8
	Widowed	30	7.5
	Married	24	6.0
Parent alive	Both dead	111	27.7
	Father only alive	102	25.5
	Both mother and father are alive	95	23.8
	Mother only alive	92	23
Monthly income in Eth Birr	500–1500	28	7
	1501–3000	200	50
	> 3000	172	43

**shows religion such as Catholic, Wakefata

### Awareness and of HIV self-testing and behavioral characteristics of female sex workers

In terms of HIV self-testing awareness among those who get tested, 97 (65.5%) had poor knowledge about HIV self-testing, but among those using HIV self-testing in total, 51 (34.5%) had good knowledge about HIV self-testing. The study showed that, 84 (21%) FSWs had their first sexual encounter under 18 years old. About 65 (16.3%) sex workers engaged in sex for less than 5 years, but 335 (83.8%) engaged in it for more than 5 years. Almost all 98.7% of them collected test kits from the navigators. Majority of them 335 (84%) were substance users.

The magnitude of HIV self-testing among FSWs was 148 (37% [95% CI: 32, 42]. Those who didn’t uptake the test mentioned that they didn’t get the offer to be tested. Of the 148 (37%) participants who had self-tested: 127 (85.8%) did that at their workplace. All the respondents reported the use of a condom during their sexual intercourse with their clients (Table 2).

**Table 2:** Behavioral characteristics of female sex workers at Waliso town, Ethiopia, 2024

Variables	Category	Frequency	Percent (%)
Age of first sexual debut	< 18 years	84	21
	≥ 18 years	316	79
Where did you use the HIVST kit	At their workplace	127	85.8
	At a friend’s house	13	8.8
	At the health facility	7	4.7
	At my family’s house	1	0.7
Who did you help interpret the results	Peer navigator	146	98.7
	Health care provider	2	1.3
Where do you collect HIV self-test kits	Peer navigator	127	85.8
	Health centers	21	14.2
Did you ask to share your client’s HIV status before engaging in sex?	Never	302	75.5
	Seldom	78	19.5
	Sometimes	7	1.8
	Often	6	1.5
	Always	7	1.8

### Factors associated with HIV Self-Testing

In the bivariable analysis, educational status (*p*-value = 0.00), marital status (*p*-value = 0.00), parent both dead (*p*-value = 0.01), monthly income (*p*-value = 0.23), and asking partner’s HIV status before sex (*p*-value = 0.00) were found to be associated with HIV self-testing. After multivariable logistic regression analysis, educational status (being high school and above), marital status (divorced), parents (both mother and father) died, and asking partner’s HIV status before engaging in sex was significantly associated with HIV self-testing (*p*-value < 0.05) (Table 3).

**Table 3:** Multiple logistics regression analysis showing significant factors associated with HIV self-testing among female sex workers at Waliso town, central Ethiopia, 2024

Variables	Category		Use HIVST		COR 95%CI	AOR 95%CI	*P*-value
			Yes, n (%)	No, n (%)			
Education	Unable to read and write		7(1.75)	16(4%)	Ref.	Ref	0.00
	Able to read and write		15(3.75)	58 (14.5)	0.6(0.21,1.7)	0.94(0.29,3.1)	0.91
	Primary education		69 (17.3)	160 (40)	0.99(0.39,2.5)	1.34(0.46,3.9)	0.59
	Secondary school and above		57 (14.25)	18 (4.5)	7.24(2.8,20.4)	7.6(2.36,24.7)	0.00*
Marital status	Single		62 (15.5)	129(32.3)	Ref.	Ref.	0.01
	Married		6(1.5)	18(4.5)	0.7(0.26,1.8)	0.75(0.26,2.2)	0.56
	Divorced		74(18.5)	81(20.25)	1.9(1.23,2.9)	2.1(1.23,3.6)	0.01*
	Widowed		6 (1.5)	24(6)	0.52(0.2,1.34)	0.58(0.2,1.76)	0.33
Parent alive	Both parents alive		33 (8.25)	62 (15.5)	Ref.	Ref.	0.00
	Mother only alive		22(5.5)	70(17.5)	0.6(0.3,1.2)	0.87(0.4,1.77)	0.69
	Father only alive		35(8.75)	67(16.75)	0.98(0.55,1.77)	1.2(0.6,2.34)	0.61
	Both parents dead		58(14.5)	53(13.25)	2.1(1.17,3.6)	2.72(1.4,5.28)	0.00*
Ask client`s HIV status before sex		Never	134(33.5)	168(42)	Ref.	Ref.	0.00
		Seldom	9(2.25)	69 (1.73)	0.16(0.08,0.34)	0.17(0.1,0.37)	0.00*
		Sometimes	1(0.25)	6(1.5)	0.21(0.03,1.76)	0.41(0.04,3.8)	0.43
		Often	1(0.25)	5(1.25)	0.25(0.03,2.17)	0.19(0.02,2.4)	0.20
		Always	3(0.75)	4(1.0)	0.94(0.21,4.27)	0.49(0.095,2.6)	0.41

*P*-value < 0.05* showing statistically significant COR: Crude Odds ratio AOR; Adjusted Odds ratio CI: Confidence interval

## Discussion

This study provides an important insights of HIV self-test and its predictors among Key Population (KP), female sex workers, in Waliso town, central Ethiopia. The finding of this study showed that the prevalence of HIV self-testing among female sex workers was 37% (95%CI: 32, 42%). This is lower than the finding from Ethiopia (59.3%) [11], South Africa (64%) [15], Senegal (74.5%) [16], and Brazil (39.3%) [17]. The magnitude in this study is below the national goal which is 95% based on UNAIDS 95–95 targets [1]. The difference may be due to variations in the study area and population characteristics, including lower-risk behaviors, effective awareness creation, and reduced population mobility, with the area being less of a population destination compared with other regions.

The odds of HIV self-testing were 7.6 times higher for female sex workers who attended secondary school and higher than for those who only completed primary school and were lower. According to research done in rural Malawi, women who had completed more education also had a greater rate of HIV self-testing [18]. Similarly, a finding from Uganda showed that teenagers with greater levels of education had a higher likelihood of having ever tested than those who didn`t attend formal education [19]. This might be because educated individuals are more knowledgeable about the benefits of HIV self-testing and have better recognition of the importance of knowing one`s HIV status, and lower levels of education correlate with less knowledge of HIV infection and lower uptake of HIV services.

Female sex workers who were divorced were two times more likely to utilize HIVST as compared to those who were single. This might be due to the fact that they may have many sexual partners and consider themselves exposed to the risk of infection. According to the WHO HIV self-testing strategic framework, these people are at high risk, and testing needs to be done [6].

Female sex workers whose both parents died were three times more likely to utilize HIVST than women living with their parents. According to earlier research, FSWs who were homeless had a higher likelihood of using HIVS than women who were living with their partners or families [20]. According to a Hong Kong study, stigma is a bigger problem for FSWs living with their families than for those living alone when it comes to getting HIV/STD health care [21]. This could be because women who live with their families are more likely to be accepted under the AIDS-related taboo than women who lost their families. Additionally, those who lose their families might participate in risky behaviors and have more risky sex; these circumstances could encourage them to get tested for HIV.

Respondents who were asked to share their client’s HIV status before engaging in sex were 83% less likely to utilize HIVST compared with their counterparts. It is necessary to address stigma and accessibility concerns in order to raise HIVST among key population in Ethiopia. To reduce stigma and encourage HIV testing, trained peer navigators should facilitate open discussions and address misconceptions. HIV self-testing kits tailored for FSWs are widely available free of charge or with discounted price. Ensuring confidentiality and privacy for FSWs and their partners is crucial to fostering trust and encouraging key populations in testing. A study conducted in Ethiopia revealed that HIV risk behavior was reported mostly among patients who did not disclose their HIV status to sex partners [22]. Owing to legal constraints, female prostitutes are willing to inquire about the HIV status of their clients. Evidence indicates that many prostitutes believe they have greater authority to mandate the use of condoms and to refuse to engage in sexual activity without one [23]. Most FSWs never inquire about their HIV status before having intercourse. Comparable findings were noted in Togo, where a small percentage of FSWs knew their partner’s status [24].

A limitation of the study is that it is cross-sectional, meaning it cannot establish cause-and-effect relationships and only shows associations between variables at a single point in time. Besides, there was a limited number of published articles on the subject to compare our findings with, specifically, the factors associated with HIV self-testing. On the other hand, there might be the possibility of recall bias for knowledge-related questions, which was addressed by designing good approaches of probing. During the implementation of the HIV self-testing program, there were many best practices and challenges. Stigma was reduced through discussion and clarification of misconceptions with trained peer navigators. Moreover, detailed awareness was given on maintaining the confidentiality and privacy of FSWs and their partners. Test kits were also provided free of charge to increase uptake. There were also robust referral mechanisms to ensure that people with positive results access medical care and counseling and that those who tested positive were quickly connected to care and treatment. However, there were challenges among those who self-tested positive to receive confirmatory testing, as required by the national testing algorithm. Some people were also preferring not to be aware of their position because they fear the consequences and that was solved through counseling and awareness.

## Conclusion

This study revealed that HIVST uptake among FSWs is considerably lower than the national requirement that was planned to be 95% based on UNAIDS 95-95-95 goals. Higher educational status, marital status, both parents’ deaths, and asking to share partner status before engaging in sex were significantly associated factors with HIVST uptake. Hence, HIV prevention programs should consider developing novel and evidence-based programs to improve the accessibility of HIV testing to FSWs and improve community-based services including awareness creation.

## Data Availability

The dataset(s) supporting the conclusions of this article is(are) included within the article.

## Abbreviations

AOR: Adjusted odds ratio
FSW: Female sex workers
HIVST: Human immunodeficiency virus self-testing
UNAIDS: United nations programme on HIV/AIDS
WHO: World health organization

## References

[1] UNAIDS, Global HIV. & AIDS statistics — 2024 fact sheet.2024. https://www.unaids.org/en/resources/fact-sheet

[2] UNAIDS, Global, UNAIDS. AIDS strategy 2021–2026. End inequalities; End AIDS. 2021. Available at: https://www.unaids.org/en/resources/documents/2021/2021-2026-global-AIDS-strategy (Last accessed 07 August 2024).

[3] Shava E, Manyake K, Mdluli C, Maribe K, Monnapula N, Nkomo B, Mosepele M, Moyo S, Mmalane M, Bärnighausen T, Makhema J, Bogart LM, Lockman S. Acceptability of oral HIV self-testing among female sex workers in Gaborone, Botswana. PLoS ONE. 2020;15(7):e0236052. 10.1371/journal.pone.0236052. PMID: 32716966; PMCID: PMC7384658. 32716966 10.1371/journal.pone.0236052 PMC7384658

[4] WHO. Guideline on HIV Self-Testing and Partner Notification. 2016. Geneva, Switzerland. http://www.who.int/hiv/pub/vct/en/

[5] : World Health Organization HIV/AIDS, Self-Testing HIV. 2019. [(accessed on 107 August 2024)]; Available:https://www.who.int/publications/i/item/WHO-CDS-HIV-19.36).

[6] FH360. HIV self-testing operational guide. For the planning, implementation, monitoring and reporting of HIV self-testing. Accesssed 07 August 2024. www.fhi360.org.

[7] Surratt HL, O’Grady CL, Kurtz SP, Buttram ME, Levi-Minzi MA. HIV testing and engagement in care among highly vulnerable female sex workers: implications for treatment as prevention models. J Health Care Poor Underserved. 2014;25(3):1360–78. 25130245 10.1353/hpu.2014.0113 PMC4137460

[8] Wood BR, Ballenger C, Stekler JD. Arguments for and against HIV self-testing. HIV AIDS (Auckl). 2014;6:117– 26. doi: 10.2147/HIV.S49083. PMID: 25114592; PMCID: PMC4126574. 10.2147/HIV.S49083 PMC4126574 25114592

[9] Harichund C, Moshabela M. Acceptability of HIV Self-Testing in Sub-Saharan Africa: scoping study. AIDS Behav. 2018;22(2):560–8. 28699017 10.1007/s10461-017-1848-9 PMC5764831

[10] Ben Moussa A, Belhiba O, Hajouji FZ, El Kettani A, Youbi M, Alami K et al. Acceptability and usability of oral fluid-based HIV self-testing among female sex workers and men who have sex with men in Morocco. BMC Public Health. 2022;22(1):1–9. Available from: 10.1186/s12889-022-14632-5 10.1186/s12889-022-14632-5 PMC9720927 36471285

[11] Eskezia BN, Tafere Y, Aschale A, Moges NA. Uptake of HIV Self-Testing and associated factors among female sex workers at Non-Governmental HIV testing facilities in Debre Markos and Bahir Dar towns, Northwest Ethiopia, 2022. HIV/AIDS -. Res Palliat Care. 2023;15(June):279–91. 10.2147/HIV.S385526 PMC10256570 37303864

[12] Mshweshwe-Pakela NT, Mabuto T, Shankland L, Fischer A, Tsukudu D, Hoffmann CJ. Digitally supported HIV self-testing increases facility-based HIV testing capacity in Ekurhuleni, South Africa. South Afr J HIV Med. 2022;23(1):1352. 10.4102/sajhivmed.v23i1.1352. PMID: 35923609; PMCID: PMC9257703. 35923609 10.4102/sajhivmed.v23i1.1352 PMC9257703

[13] Central Statistical Agency. The 2007 National census preliminary report for Ethiopia. Addis Ababa, Ethiopia: Central Statistical Agency; 2008.

[14] Von Elm E, Altman DG, Egger M, Pocock SJ, Gotzsche PC, Vandenbroucke JP. The strengthening the reporting of observational studies in epidemiology (STROBE) statement: guidelines for reporting observational studies. Lancet. 2007;370(9596):1453–7. pmid:18064739. 18064739 10.1016/S0140-6736(07)61602-X

[15] Mthiyane HR, Makatini Z, Tsukulu R, Jeena R, Mutloane M, Giddings D et al. HIV self-testing: a cross-sectional survey conducted among students at a tertiary institution in Johannesburg, South Africa in 2020. 2023;14:115–9. 10.4081/jphia.2023.2227 PMC10334437 37441117

[16] Lakhe NA, Diallo Mbaye K, Sylla K, Ndour CT. HIV screening in men and women in Senegal: coverage and associated factors; analysis of the 2017 demographic and health survey. BMC Infect Dis. 2019;20(1):1. 10.1186/s12879-019-4717-5. PMID: 31892320; PMCID: PMC6938616. 31892320 10.1186/s12879-019-4717-5 PMC6938616

[17] Brito AM, Szwarcwald CL, Damacena GN, Dourado IC, Brazilian FSW, Group. HIV testing coverage among female sex workers, Brazil, 2016. Rev Bras Epidemiol. 2019;22Suppl 1(Suppl 1):e190006. Published 2019 Sep 26. 10.1590/1980-549720190006.supl.1 10.1590/1980-549720190006.supl.1 31576982

[18] Indravudh PP, Hensen B, Nzawa R, et al. Who is reached by HIV Self-Testing? Individual factors associated with Self-Testing within a Community-Based program in rural Malawi. J Acquir Immune Defic Syndr. 2020;85(2):165–73. 10.1097/QAI.0000000000002412 32501815 10.1097/QAI.0000000000002412 PMC7611247

[19] Atuhaire L, Shumba CS, Mapahla L, Maposa I, Nyasulu PS. Factors associated with adherence to HIV testing guidelines among HIV-negative female sex workers in Kampala, Uganda. IJID Reg. 2022;4:25–32. 10.1016/j.ijregi.2022.05.008. Published 2022 May 28. 36093368 10.1016/j.ijregi.2022.05.008 PMC9453214

[20] Ahmadi S, Khezri M, Roshanfekr P, et al. HIV testing and its associated factors among street-based female sex workers in Iran: results of a National rapid assessment and response survey. Subst Abuse Treat Prev Policy. 2021;16(1):43. 10.1186/s13011-021-00382-x. Published 2021 May 17. 34001164 10.1186/s13011-021-00382-x PMC8130331

[21] Ma H, Loke AY. A qualitative study into female sex workers’ experience of stigma in the health care setting in Hong Kong. Int J Equity Health. 2019;18(1):175. 10.1186/s12939-019-1084-1. Published 2019 Nov 14. 31727157 10.1186/s12939-019-1084-1 PMC6857210

[22] Damtie Y, Kefale B, Yalew M, et al. HIV risk behavior and associated factors among people living with HIV/AIDS in Ethiopia: A systematic review and meta-analysis. PLoS ONE. 2022;17(7):e0269304. 10.1371/journal.pone.0269304. Published 2022 Jul 28. 35901123 10.1371/journal.pone.0269304 PMC9333449

[23] Lancaster KE, Cernigliaro D, Zulliger R, Fleming PF. HIV care and treatment experiences among female sex workers living with HIV in sub-Saharan Africa: A systematic review. Afr J AIDS Res. 2016;15(4):377–86. PMID: 27974017; PMCID: PMC5541376. 27974017 10.2989/16085906.2016.1255652 PMC5541376

[24] Bitty-Anderson AM, Gbeasor-Komlanvi FA, Tchankoni MK et al. HIV prevalence and risk behaviors among female sex workers in Togo in 2017: a cross-sectional national study. Arch Public Health. 2022;80(1):92. Published 2022 Mar 24. 10.1186/s13690-022-00851-0 10.1186/s13690-022-00851-0 PMC8943989 35331303

